# Recent Advances in ZnO-Based Carbon Monoxide Sensors: Role of Doping

**DOI:** 10.3390/s21134425

**Published:** 2021-06-28

**Authors:** Ana María Pineda-Reyes, María R. Herrera-Rivera, Hugo Rojas-Chávez, Heriberto Cruz-Martínez, Dora I. Medina

**Affiliations:** 1 Laboratorio de Investigación y Posgrado en Tecnología Farmacéutica, Facultad de Estudios Superiores Cuautitlán, Universidad Nacional Autónoma de México, Av. 1o. de Mayo S/N, Cuautitlán Izcalli, Estado de Mexico 54740, Mexico; anamaria.pineda.reyes@gmail.com; 2 Tecnologico de Monterrey, Escuela de Ingeniería y Ciencias, Ave. Eugenio Garza Sada 2501, Monterrey, Nuevo León 64849, Mexico; maria.herrera@tec.mx; 3 Tecnológico Nacional de México, Instituto Tecnológico de Tláhuac II, Camino Real 625, Tláhuac, Ciudad de Mexico 13508, Mexico; rojas_hugo@ittlahuac2.edu.mx; 4 Tecnológico Nacional de México, Instituto Tecnológico del Valle de Etla, Abasolo S/N, Barrio del Agua Buena, Santiago Suchilquitongo, Oaxaca 68230, Mexico; 5 Tecnologico de Monterrey, School of Engineering and Sciences, Atizapan de Zaragoza, Estado de Mexico 52926, Mexico

**Keywords:** ZO doping, CO gas, ZnO sensors, sensitivity, selectivity, density functional theory

## Abstract

Monitoring and detecting carbon monoxide (CO) are critical because this gas is toxic and harmful to the ecosystem. In this respect, designing high-performance gas sensors for CO detection is necessary. Zinc oxide-based materials are promising for use as CO sensors, owing to their good sensing response, electrical performance, cost-effectiveness, long-term stability, low power consumption, ease of manufacturing, chemical stability, and non-toxicity. Nevertheless, further progress in gas sensing requires improving the selectivity and sensitivity, and lowering the operating temperature. Recently, different strategies have been implemented to improve the sensitivity and selectivity of ZnO to CO, highlighting the doping of ZnO. Many studies concluded that doped ZnO demonstrates better sensing properties than those of undoped ZnO in detecting CO. Therefore, in this review, we analyze and discuss, in detail, the recent advances in doped ZnO for CO sensing applications. First, experimental studies on ZnO doped with transition metals, boron group elements, and alkaline earth metals as CO sensors are comprehensively reviewed. We then focused on analyzing theoretical and combined experimental–theoretical studies. Finally, we present the conclusions and some perspectives for future investigations in the context of advancements in CO sensing using doped ZnO, which include room-temperature gas sensing.

## 1. Introduction

Carbon monoxide (CO) is a pollutant gas that can be toxic if inhaled in large amounts, and its release outdoors contributes to air pollution [1,2,3]. Exposure to CO levels above 70 ppm can cause headaches, dizziness, disorientation, and fatigue [4,5,6]. In this context, prolonged exposure to higher CO levels (150–200 ppm) causes prejudicial cardiopulmonary events, which may lead to death; however, it has been reported that in certain cases, short exposure to levels of approximately 50 ppm can also be fatal [5,6,7,8]. It is worth keeping in mind that CO is absorbed into the blood through the lungs, forming carboxyhemoglobin, reducing the concentration of oxygen in the blood, preventing the binding to hemoglobin [9]. Considered as a silent killer [10,11], CO is an odorless, colorless, and tasteless gas [11,12] produced by the incomplete combustion of fossil fuels [4,13,14], automobile exhaust emissions [4,15,16,17], domestic fuel burning [18], gas fires [14], agriculture product burning, volcanic activity [18], industrial exhaust emissions [16], and fault monitoring in large power equipment [19] and carbon-containing compounds, such as domestic appliances [4,14] and coal mines [7,20]. Lately, CO emissions have been increasing, owing to accidental leakage associated with technological advances in electrical appliances [21], greater environmental pollution, industrialization activities [18], and incomplete combustion of wood, crude oil, and natural gas derivatives with other pollutants [10]. Its detection and monitoring are of great interest to prevent it from going unnoticed because of its characteristics mentioned earlier [15,21]. Therefore, designing high-performance gas sensors for detecting this highly toxic gas is necessary. According to Hulanicki et al., a gas sensor can be defined as a device that detects the presence of volatile substances in the vapor phase, both qualitatively (kind) and quantitatively (concentration), in a specific volume [22]. Following this definition, in some cases, various hazardous gases are detected successfully and efficiently via the parameters shown in Table 1 [22,23].

Metal oxide semiconductors (MOSs) have been successfully used to detect CO gas. MOSs have low cost, high sensitivity [24,25,26,27,28,29,30], convenient operation, a rapid response and recovery time, high physical and chemical stabilities [22], excellent electrical performance [16], and a simple and portable design [2]. Among various MOSs, those based on ZnO have become widely manufactured and utilized because of their outstanding characteristics, such as a bandgap around 3.4 eV at room temperature [31,32,33], high optical transparency in the visible region (>80%), n-type conductivity, and high exciton binding energy in the order of 60 meV [31,34]. They also have a versatile morphology, rendering them suitable for usage in different industrial applications, including gas sensor devices, by their potential to detect various toxic gases. It is worth pointing out that though ZnO belongs to wurtzite, with a hexagonal crystal structure and space group of P63 mc, it has the following two other possible crystal structures: cubic zinc blende-type and rock salt structure [32]. However, ZnO highly tends to crystallize in the wurtzite-type structure. Concerning its morphology, ZnO has been obtained as nanostructures, which include nanoneedles, nanosticks, nanocouples, nanoflakes, nanosprings, nanotubes, nanorods, and nanowires [31,34,35], as well as porous and dense films [36]; as gas sensing performance is mainly dependent on the morphology of the material. Significant achievements have been attained in the fabrication of gas sensors based on ZnO structures, including biosensors [16,30,36,37,38,39,40,41]. Nevertheless, further research is needed to improve CO detection at low operating temperatures (e.g., room temperature) and with excellent selectivity to differentiate two gases with similar behaviors, considering the effect of the relative humidity on the gas sensing performance [38,42,43].

Undoubtedly, to date, ZnO is a promising candidate for detecting CO gas; it offers good sensitivity and selectivity, as well as a high surface area for excellent gas sensor response and adsorption sites. For instance, the porous morphology of ZnO increases its surface area, which, along with high electron mobility, excellent electrical properties, and adsorption sites, allow an excellent gas sensor response [3,7,12,18,28,29,30,43,44]. However, further research is needed to improve the selectivity and sensitivity for CO gas detection. In this sense, increasing the resistance to humidity and working at lower temperatures should be given more attention [10,16]. Consequently, several strategies have been implemented to optimize the sensing properties of ZnO. These include different synthesis methods, doping, surface modification, coatings, UV activation, functionalization, inclusion of carbonaceous nanomaterials, use of nanocomposites, post-treatments, and high-energy irradiation methods [16,29,40,43]. Doping is the process that involves a metal atom replacement into the crystal lattice of a metal oxide, and due to a small addition (usually at. %) of foreign atoms several properties are modified, e.g., ZnO doping demonstrates favorable advantages for improving the gas sensor performance. It is well-known that doping during synthesis and deposition processes affects those properties that are significant for gas sensing applications. For instance, doped ZnO improves the detection limits and sensing performance compared with those of pure ZnO. Furthermore, parameters such as the selectivity, sensitivity, response time, and stability of the gas sensors are improved by adding different dopant elements. To date, various sensors have been designed and fabricated using doped ZnO to improve the operating conditions at low temperatures, and retain high sensitivity at low concentrations, good selectivity, and fast response/recovery times, in addition to good repeatability and stability [6,29,45,46,47,48,49,50,51,52,53].

Because of the critical role of doped ZnO in gas sensor design, numerous experimental, theoretical, and combined experimental–theoretical studies have been conducted to investigate novel doped ZnO sensors used in CO gas detection. These illustrate doping as an effective approach for improving the CO detection capacity with respect to undoped samples. Notwithstanding the sizeable number of studies published, there is a lack of detailed and critical reviews on the current progress in experimental, theoretical, and combined experimental–theoretical studies around the design of doped ZnO toward CO gas detection. Therefore, in this review, we cover recent reports regarding the CO detection ability of doped ZnO. First, experimental studies on ZnO doped with transition metals, boron group elements, and alkaline earth metals as CO sensors are comprehensively reviewed. We then focused on analyzing theoretical and combined experimental–theoretical studies. Finally, we present conclusions, and the current challenges are outlined.

## 2. Experimental Studies

ZnO is an MOS that has been extensively studied as a gas sensor. However, several research groups are investigating the potential of adding elements in the crystal lattice of ZnO as doping materials. The research outcomes revealed that the doping elements change the ZnO structure. For example, doping elements decrease the crystallite size, increase the crystallinity, and modify the ZnO morphology [54,55,56,57]. The structural and morphological changes increase the surface-to-volume ratio, creating a more active center at the grain boundaries [58]. Consequently, the sensitivity [59], response/recovery time, selectivity, and working temperature [60] are improved, which, in terms of practical applications, are desirable. Therefore, doping is a viable way of improving the ZnO sensing properties [61]. Given this, various types of doped ZnO gas sensors have been developed [62].

### 2.1. Transition Metals

In recent years, various transition metals have been explored for doping ZnO particles and improving their sensitivity to CO gas. The sensing properties of the ZnO structures are remarkably enhanced when they are doped with transition metals compared with those of pure ZnO, which has limited sensitivity to chemically stable gases. Transition metals, such as Cu, Mn, Ni, Pt, and Fe, have been used as dopants for CO sensors using different synthesis routes (e.g., hydrothermal, sol-gel, sputtering, and in situ reduction). Although so many techniques have been reported in the last years to obtain ZnO, here, we strictly focused on the punctual synthetic approaches that provide a general idea about some synthetic methods. A systematic introduction to the methods of synthesizing ZnO is reported in the literature [63].

**Figure 1:**
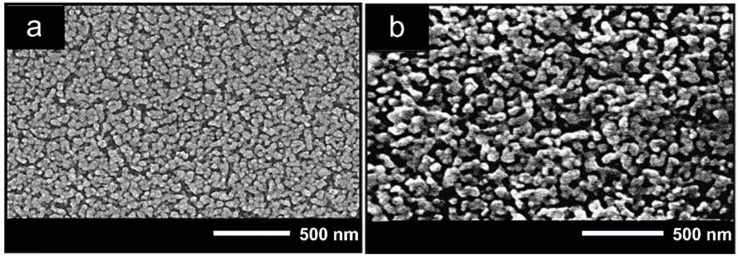
SEM images of undoped ZnO (**a**) and Mn-doped ZnO (**b**) nanostructures. Modified from Reference [65].

A key observation is that nanostructured Cu-doped ZnO sensors were obtained by chemical synthesis, using a parallel reaction station with a Cu concentration of 1 at. %, and were manufactured in the form of pellets [64]. The Cu-doped ZnO sensor evidenced improved CO gas detection properties compared with those of the undoped material. It is worth highlighting that ZnO powders showed a change in their morphology due to doping. Moreover, the Cu-doped ZnO sensor presented an increase in sensitivity (60%) compared with that of the undoped ZnO sensor (33%) at 100 ppm of CO gas. Another interesting feature was the optimal operating temperature, as follows: 95 °C for the Cu-doped ZnO sensors and 115 °C for the undoped ZnO sensor. The decrease in the working temperature was associated with the thermal energy, that is, the doped sensors require less thermal energy to excite the electrons on the conduction band. In this context, the high sensitivity of the Cu-doped ZnO sensor was also attributed to the fact that Cu sites improve the adsorption and reaction of CO with oxygen species [64]. In the same study [64], Karaduman et al. obtained ZnO nanostructures doped with Mn at conditions similar to those of Cu. Although Mn modified the ZnO shape and size, as shown in Figure 1a,b, it only achieved a slight increment in CO gas sensitivity (40%) compared with that of the undoped ZnO (33%). Accordingly, the results obtained by Karaduman et al. demonstrated that Cu-doped ZnO sensors, compared with the Mn-doped and undoped ZnO sensors, are better when sensitivity is a concern [64].

**Figure 2:**
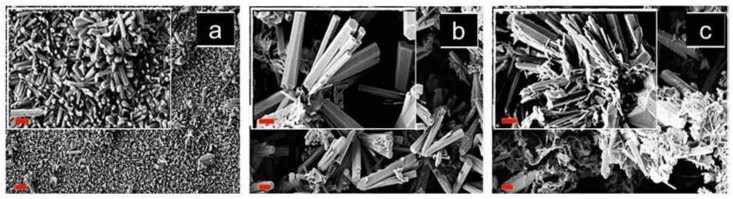
SEM images (scale bars: 2 μm) of the powder sample. (**a**) Undoped ZnO, (**b**) Ni-doped ZnO (5 at. %), and (**c**) Ni-doped ZnO (10 at. %). Note that in insets scale bars: 1 μm. Modified from Reference [8].

In another study, Shirage et al. investigated and constructed a sensor using ZnO nanorods doped with Ni (5 and 10 at. %) [8]. A newly developed chemoresistive sensor was prepared using a wet chemical synthesis, which exhibited CO gas sensitivity and selectivity. The Ni-doped ZnO (5 at. %) sensor demonstrated the highest response to CO compared with those of the undoped ZnO and Ni-doped ZnO (10 at. %) sensors at 250 °C. The sensitivity values at 200 ppm were approximately 1.5, 5, and 2.75 *R*_0_/*R* (*R*_0_ represents the reference resistance in the air atmosphere, and *R* represents the resistance in the target gas atmosphere) for undoped ZnO, Ni-doped ZnO (5 at. %), and Ni-doped ZnO (10 at. %), respectively. It is important to notice that the Ni-doped ZnO (5 at. %) sensor at 250 °C had a rapid response time (15 s) and a recovery time of less than 90 s. Figure 2 shows the scanning electron microscopy images of the undoped and Ni-doped ZnO samples. These images revealed changes in the ZnO morphology, caused by Ni doping. According to Shirage et al., the good sensitivity of the Ni-doped ZnO (5 at. %) sensor is attributed to its morphological and microstructural characteristics [8].

Recently, Wang et al. synthesized Pt-doped ZnO nanosheets with various concentrations of Pt (0, 0.25, 0.50, 0.75, and 1.00 at. %), using the hydrothermal method [59]. The sensor response to 50 ppm of CO gas of the Pt-doped ZnO (0.50 at. %) was 3.57 *R*_0_/*R*. Also, the sensor showed short response and recovery times of 6 and 19 s, respectively. The short response and recovery times were attributed to the catalytic effect of Pt. In addition, a decrease in the operating temperature (from 210 °C to 180 °C) was observed compared with the undoped ZnO sensor. This decrease in the operating temperature is associated with the addition of Pt in the ZnO, because the catalyst decreased the activation energy of gas chemisorption [66].

Another approach has been investigated lately by researchers; it is the use of Fe as a doping element. In this sense, Kumar et al. fabricated a gas sensor using Fe-doped ZnO thin films on a conducting glass, using the spin-coating method [67]. This device demonstrated significantly better CO gas detection sensitivity, excellent gas response, and better stability than those of the undoped ZnO. This study proved the linear dependence relationship between the sensor response and the operating temperature. However, in the case of the pure ZnO gas sensor, the sensing response (4.25) increased up to 400 °C; subsequently, the sensitivity decreased. In contrast, for the Fe-doped ZnO gas sensor, the sensitivity (5.75) increased up to 500 °C and then decreased. The optimal operating temperature was 150 °C. In addition, the response and recovery times at 150 °C of the Fe-doped ZnO thin films were shorter than those of the undoped ZnO thin films. Therefore, the researchers confirmed that the Fe-doped ZnO thin films demonstrated sensitivity higher than those of the undoped ZnO thin films, as the electrical conductivity was higher in the Fe-doped ZnO than that in the undoped ZnO. Although Fe has an ionic radius close to that of Zn, which allows retention of the lattice structure and favors its incorporation into the lattice (due to the close electronegativity), researchers have observed a reduction in the crystallinity levels because of the ionic radii contraction of Fe^3+^ to slightly less than the ionic radii of Zn^2+^ [67].

Co-doped Fe and Ni zinc oxide (ZnO:Fe:Ni) gas sensors have also been reported to detect CO gas. For instance, Jayaraman et al. reported the CO gas sensing response of ZnO:Fe:Ni films [68]. In this study, Co-doped ZnO:Fe:Ni thin films were deposited on glass substrates using the ultrasonic spray pyrolysis technique. Fe and Ni, with different concentrations (1.5, 2.5, and 5 at. %) with respect to the Zn content, were used as dopants. The undoped ZnO films show a hexagonal wurtzite structure, but a low-intensity peak appeared in the co-doped ZnO films. This extra peak corresponds to magnetite in its face-centered cubic phase. The undoped ZnO film showed irregular grains formed by stacked planar hexagonal-shaped subgrains. For the ZnO:Fe:Ni films, the grain shape varies, as follows: orthorhombic shapes (1.5 at. %), no homogeneous grains (2.5 at. %), and rock shapes (5 at. %) are obtained. The optimal gas sensing parameters were a gas concentration of 300 ppm and a working temperature of 300 °C. The ZnO:Fe:Ni film at 1.5 at. % presented the best sensitivity (~10). Jayaraman et al. demonstrated that low doping concentrations improve the CO gas sensing response, which favors the grain size increment, creating agglomerates and suturing the surface of the ZnO:Fe:Ni films [68].

Generally, doping elements markedly improve the sensitivity of ZnO sensors. The Ni-doped ZnO sensor showed the best response to CO gas. The sensitivity of the undoped ZnO was 1.5, whereas that for the Ni-doped ZnO at 5 at. % was 5 [8]. The second-best doping element was Pt. The sensitivity of the Pt-doped ZnO sensor at 0.5 at. % was 3.75 [59]. However, it should be noted that CO doping is an excellent option for improving the sensor response in the presence of CO. A ZnO:Fe:Ni sensor, with a response of 10, has been reported, and the response is considered high compared with that of the pure ZnO sensor [68].

### 2.2. Boron Group

**Figure 3:**
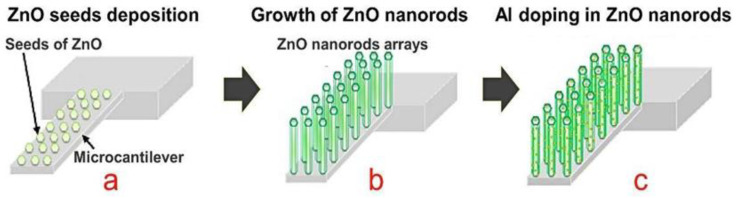
Growth process of Al-doped ZnO nanorods on microcantilever consisting of (**a**) ZnO seeds deposition; (**b**) the growth of ZnO nanorods; (**c**) Al doping in ZnO nanorods using radio frequency sputtering. Modified from Reference [69].

Other metals have also been reported in the literature, as dopants of ZnO are boron group metals. However, only a few research studies on these doping elements, such as Al, In, and Ga, have been reported. ZnO particles doped with these metals showed improved sensing properties, such as selectivity and response and recovery times. Some synthesis routes used to dope ZnO nanoparticles with Al, In, or Ga are the sol-gel, hydrothermal, spin-coating, spray pyrolysis, and microemulsion techniques. For instance, Nuryadi et al. evaluated the CO sensing properties of Al-doped ZnO nanorods grown directly on a microcantilever (MC), using the hydrothermal method (Figure 3). Sensitivity measurements were performed by changing the resonance frequency of the sensors based on doped and undoped ZnO. When the CO gas flow was 200 mL min^−1^, a change in the resonance frequency of the doped sensor was observed at approximately 29.3–30.4 kHz. In contrast, the undoped sensor showed no response in the presence of the CO gas [69]. In another study [63], the CO gas sensitivity of this sensor was evaluated under relative humidity levels. A change in the resonance frequency, approximately between 30.55 and 30.57 kHz of the sensor doped with Al at a CO gas flow of 100 mL min^−1^, was observed. Therefore, it can be concluded that Al-doped ZnO sensors require greater attention to improve their sensitivity at lower CO flow rates in the presence of low relative humidity levels [70].

**Figure 4:**
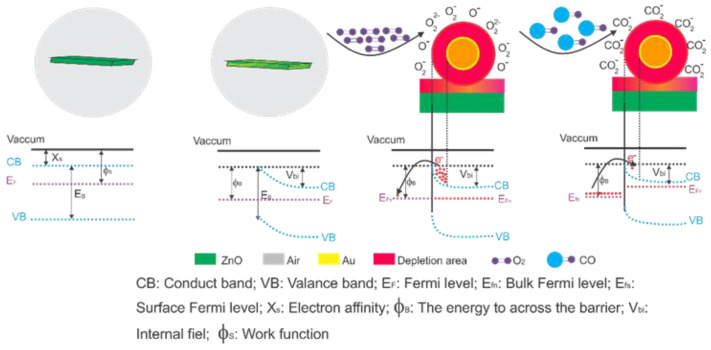
Gas sensing mechanism and energy band of zinc oxide doped before and after CO exposure nanostructures. Modified from Reference [57].

Another CO sensor based on Al-doped ZnO was designed by Jabbar et al. [71]. They used n-type and p-type porous silicon as a substrate. In their study, enhanced CO gas detection sensitivity was observed because of the liberation of electrons from the ZnO conduction band, and enhancement of the active layer of ZnO due to Al carriers. Consequently, the resistance of the sensor decreased. Notably, increasing the Al concentration increases the sensitivity from 1.4% to approximately 2.7% with respect to that of the undoped ZnO sensor at 200 °C on the n-type substrate with a CO concentration of 1000 ppm. This is attributed to the variation in the electrical phenomena present on the film surface [71], as exemplified in Figure 4 [57]. A possible mechanism of CO adsorption onto the doped ZnO surface is shown in Figure 4. A possible explanation is that the ZnO band diagram is represented as a flat band at equilibrium. Once the ZnO nanoparticles are doped, a band deflection is observed, creating a zone of electron accumulation and increasing their work function [57]. At room temperature, the film’s surface adsorbs the most stable O species (O_2_) [71]. Then, the temperature is increased to create more reactive oxygen species and facilitate the chemisorption of the CO molecules on the surface of the doped ZnO film. This is shown in the band diagram as the capture of electrons from the conduction band, which is interpreted as a change in electrical resistance [57].

In addition, Lim et al. evaluated the sensing properties of undoped and Al-doped ZnO nanorods at 5 at. %. The ZnO nanorods were synthesized using a microemulsion method. The sensitivity of the undoped ZnO nanorods presented a 50% response at an operating temperature of 300 °C. In contrast, for the Al-doped ZnO nanorods, the maximum sensitivity (60% response) was obtained at 350 °C. The electrical conductivity of ZnO increases as the concentration of aliovalent dopant carriers increases, whereas the increase in the sensors’ working temperature follows the complex exothermic adsorption of CO gas [72]. Likewise, Al has been evaluated along with Ga for doping ZnO sensors. For instance, Al-Asedy et al. prepared thin films of ZnO doped with Al and Ga at 1 at. % and 3 at. %, respectively, on the n-type Si substrate, using the spin-coated method. They reported a maximum CO gas sensitivity of 279% at an exposure time of 60 min and a working temperature of 100 °C. This point becomes particularly important because they deduced that CO doping decreased the working temperature. In contrast, the pure ZnO maintained a higher working temperature [73].

Further, Zhang et al. evaluated the sensitivity of Al-doped ZnO nanoparticles deposited on alumina substrates. Al-doped ZnO particles were synthesized with different Al concentrations (1 at. %, 2 at. %, 3 at. %, and 4 at. %) using a colloid chemistry method. The CO sensitivity response was evaluated at concentrations from 5 ppm to 80 ppm using different working temperatures from 100 °C to 300 °C. The sensing response improved with the addition of Al, with the best response at 1 at. %. This is because Al is ionized into Al^3+^ and replaces Zn^2+^, which in turn increases the electron concentration. However, if the Al concentration increases, neutral interstitial defects are formed, thereby decreasing the free electron concentration. A higher CO response for the Al-doped ZnO sensors was observed at 80 ppm and 250 °C, with response and recovery times of 15 and 7 s, respectively. Zhang et al. observed that if the working temperature is higher than 250 °C, the CO adsorption decreases, and the gas response weakens [74].

Hjiri et al. synthesized Al-doped ZnO nanoparticles (3 at. %) through the sol-gel process. The nanoparticles were deposited on alumina substrates. These sensors were evaluated for monitoring CO gas. According to Hjiri et al., the Al-doped ZnO sensor showed excellent CO gas sensitivity (80% at 50 ppm). This high sensitivity was attributed to an increase in the electrical conductivity due to the Al dopant [75].

**Figure 5:**
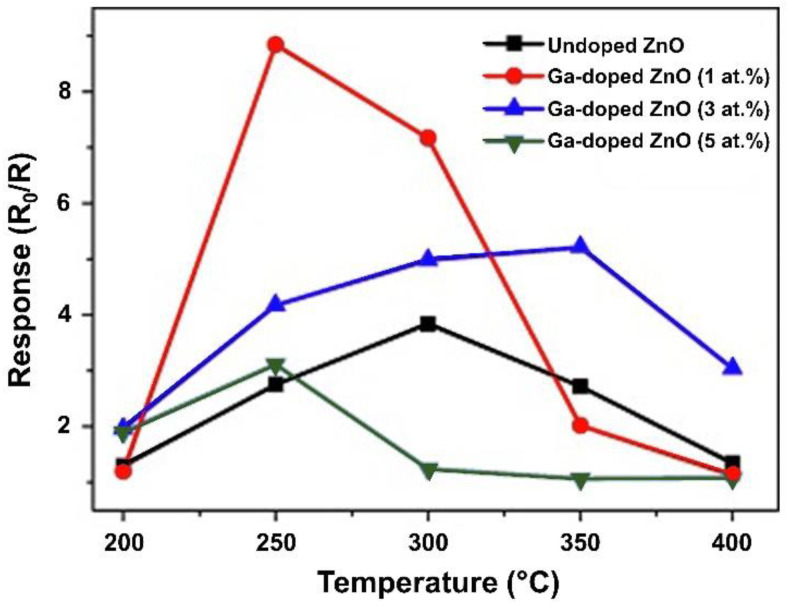
Response to 50 ppm CO of the Ga-doped ZnO nanoparticles as a function of the temperature. Ga concentration of samples correspond at 0, 1, 3 and 5 at. %. Obtained from Reference [77].

Alternatively, Ga is another promising metallic element that can be used as a dopant in ZnO-based sensors to improve CO gas detection. The incorporation of Ga (3 at. %) into the crystal lattice of ZnO notably improves the sensitivity, because it decreases the crystallite size, as reported by Hjiri et al. [76]. The sensor designed by these researchers had the highest sensitivity to 50 ppm of CO compared with that of the undoped ZnO sensor at an operating temperature of 250 °C. The increase in sensitivity is because of the decrease in the average crystallite size (approximately 49 nm) of the Ga-doped ZnO nanoparticles compared with that of the undoped ZnO nanoparticles (approximately 55 nm). This is a notable advancement compared with the undoped ZnO nanoparticles, because the reduction in crystallite size helps increase the surface area. The Ga addition improves the reaction of CO with oxygen species, which in turn improves the response and recovery times of the sensor (7 s and 16 s, respectively) at 300 °C [76]. In another work, Hjiri et al. analyzed a sensor with similar characteristics. They doped ZnO with different concentrations of Ga (1, 3, and 5 at. %) and evaluated their CO gas sensing properties. Figure 5 shows the sensitive values of the Ga-doped ZnO nanoparticles. The maximum sensitivity of the Ga-doped ZnO sensor can be observed at 250 °C (1 at. %). Thereafter, the sensitivity decreased when the operating temperature increased. A change in the morphology of the particles with increased Ga concentrations was observed [77]. The study also concluded that the solubility limit was 3 at. % Ga in ZnO. When this doping limit is exceeded, the Ga atoms distort the lattice until they cause phase segregation [77].

Another approach was reported by Dhahri et al., where In was used to dope ZnO (1, 2, 3 and 5 at. %) [78]. Interestingly, they all improved the sensitivity of the In-doped ZnO sensor under a CO gas environment in comparison with the sensitivity of the undoped ZnO sensor. This behavior has been attributed to the most active adsorption sites, assuming the substitution of the Zn^2+^ cation by In^3+^. Therefore, the In sites favored the reaction of CO with the different oxygen species. Sensors doped with 1 at. % and 2 at. % were found to be more sensitive, with shorter response and recovery times, than those with a high concentration of doping. The authors concluded that the incorporation of In in the crystal lattice changes the oxygen stoichiometry of ZnO and influences the sensing behavior of doped ZnO. Nonetheless, they suggested that doping should be performed at low concentrations (less than 2 at. %). This is because at higher dopant concentrations, the oxygen species are strongly bound to the dopant sites, and, therefore, fewer sites are available for interaction with CO [78].

Another attempt used B, which is considered a metalloid because it behaves as a metal or nonmetal. Metalloids have also been investigated as doping materials to improve the CO gas sensitivity and selectivity of ZnO structures. In this sense, undoped ZnO and B-doped ZnO (2, 4, 6, and 8 at. %) thin films were deposited on n-type and p-type silicon substrates, using the spray pyrolysis deposition method. The sensitivity increased as the B concentration increased in the B-doped ZnO thin films. This can be attributed to the electronic and structural modifications that B introduces in the B-doped ZnO thin films [79]. Among the analyzed doping elements of the B group, it is concluded that Al-doped ZnO (1 at. %) was the best doping element, with a sensing response of 11.

### 2.3. Alkaline Earth Metals

Other metals reported in the literature that enhance the sensing properties of doped ZnO are alkaline earth metals, namely, group II elements. In this respect, some alkaline earth metals, such as Ca and Sr, have been explored in the development of sensors and incorporated as active materials into the ZnO structure. In this context, Ghosh et al. successfully developed a sensor that demonstrates cross-sensitivity to CO and CO_2_ in a mixed H_2_ and CO_2_ environment. They incorporated a Ca-doped ZnO thin-film layer at 5 at. % on a piezoelectric substrate of langasite to operate at a high temperature (400 °C) through wet chemical synthesis [80]. Ghosh et al. demonstrated a predominant improvement in the cross-sensitivity to CO gas at 500 ppm. This was observed through the mass loading effect and conductivity of the thin-film coating on the substrate, because these are the sensing principles of a surface acoustic wave (SAW) sensor. The mass loading effect allowed the reduction in the resonant frequency to 6.627750 MHz in the Ca-doped ZnO thin-film sensor in comparison with 7.89 MHz for the pure langasite. The cross-sensitivity to CO had a response time of 87 s and a recovery time of 101 s, and the response was at 1.605 KHz. Comparison studies were conducted with commercial sensors that can operate at temperatures exceeding 800 °C. They observed that langasite-based SAW gas sensors are extremely beneficial because these can operate at temperatures exceeding 350 °C. They concluded that the n-type-coated Ca-doped ZnO thin film enabled an increment in the conductivity, reduced the wave velocity, and reduced the sensor resonant frequency [80]. Similar results were observed for a chemoresistive sensor with a layer of Ca-doped ZnO thin film at 5 at. % and 350 °C in the presence of CO, H_2_, and CO_2_ gases [81].

**Figure 6:**
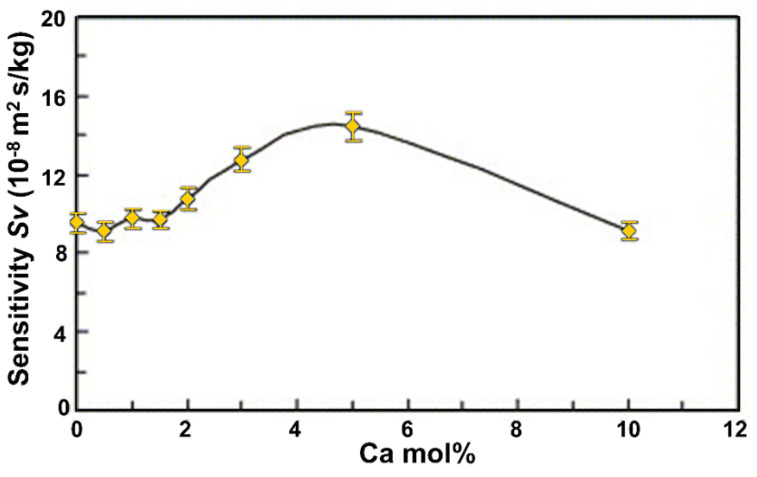
Increased sensitivity as a function of Ca dopant concentration. Obtained from Reference [83].

A recent study reported that the Ca-doped ZnO nanoparticles used in electrochemical sensors showed significant sensitivity, selectivity, and reproducibility, owing to the increased surface area in the doped ZnO [82]. Another study evaluated different concentrations of Ca (as a dopant) used to modify the crystalline structure of the ZnO films in Love wave sensors, confirming an increase in sensitivity due to the increase in the surface roughness after the addition of Ca [83]. The rough surface of the film enables physical phenomena, such as the nonlaminar movement of gas as it interacts with the surface of the Ca-doped ZnO film, facilitating the chemisorption of the CO molecule. The doping limit is 5 mol% of Ca. At higher percentages, the porosity of the surface decreases, that is, it becomes smooth again (Figure 6) [83]. In addition, other authors have reported how Ca modifies the ZnO properties when it is incorporated into the ZnO lattice (i.e., at 0 at. %, the particle size was 38 nm and the lattice constant was 3.248 Å; at 3 at. %, the particle size was 53.5 nm, and the lattice constant was 3.260 Å). A linear trend for the lattice constant was accompanied by increasing the crystallite sizes. With increasing Ca concentrations, a slight decrease in the bandgap energy is observed, owing to the increased crystallite size [84].

Shirage et al. [8] investigated and constructed a sensor using ZnO nanorods doped with Sr. The newly developed chemoresistive sensor was prepared using a wet chemical synthesis method, exhibiting sensitivity and selectivity to CO and CO_2_. As the response increased toward lower temperatures, the detection of both gases was enhanced compared with that of the undoped ZnO. They considered the incorporation of Sr in the nanorods as excellent by the presence of lattice distortion in the crystalline structure of ZnO [8].

## 3. Density Functional Theory Studies on Doped ZnO-Based CO Sensors

At the experimental level, the design of novel doped ZnO sensors is widely affected by the lack of rapid and economical synthesis routes that allow controlling the doping concentration in ZnO structures. Undoubtedly, such drawbacks can be overcome if sensor materials are modeled using first-principle or ab initio methods. In this context, first-principle simulations based on the density functional theory (DFT) are essential for explaining and understanding experimental results at the molecular level, or for predicting novel gas sensors [85,86,87,88]. DFT-based simulations provide relevant critical information for designing novel gas sensors. For instance, descriptors, such as the most stable adsorption sites, the adsorption energy, charge transfer, electronic modification after gas adsorption, and feasible approaches to enhancing gas adsorption or desorption, are obtained via DFT [87,88]. Because of the critical role that DFT calculations play in the design of toxic gas sensors, various DFT-based theoretical studies have been conducted to investigate novel ZnO-based gas sensors with different dopants to detect CO gas [89,90,91,92,93,94,95,96,97]; see Table 2.

**Figure 7:**
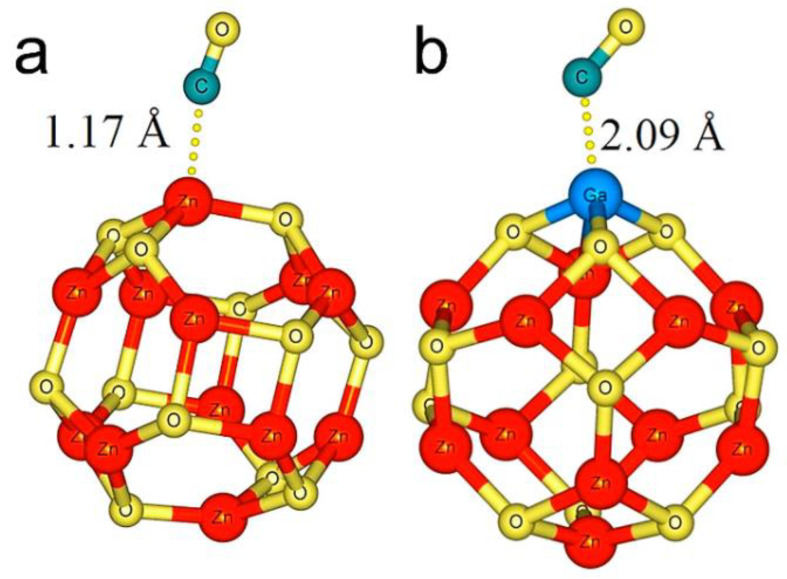
Optimized structures of CO adsorption on undoped Zn_12_O_12_ (**a**) and Ga-doped Zn_11_O_12_ (**b**) clusters. Obtained from Reference [96].

Until now, more than ten metals have been investigated as doping elements to improve the ZnO sensitivity to CO gas, among which Al is the most studied metal (Table 2). In this context, general gradient approximation, specifically Perdew–Burke–Ernzerhof (PBE), has been the most widely used method. It has also been observed that Zn_12_O_12_ clusters are the most widely used to study doped ZnO as a CO gas sensor, because they have a stable structure (Figure 7). Interestingly, several studies consider dispersion corrections in the calculations, which allow better descriptions of the interaction between CO and doped ZnO. Note that in the DFT calculations, the methodology and approaches used influence the results obtained. For example, for the Al-doped ZnO, two types of approaches were used in the calculations (triangular nanowire and slab). It is observed that the CO adsorption energy on these systems changes, showing that the parameters used in the calculations indeed influence the results obtained. According to the CO adsorption energy used to evaluate the sensor sensitivity, the doped ZnO adsorbed CO better than the undoped ZnO. It is also observed that the best doping materials are Pt, Cr, and Fe because they present the highest adsorption energy, indicating that these could deliver the best CO gas sensitivity. The excellent sensitivity of doped ZnO is attributed to its modified electronic and electrical properties compared with those of the undoped ZnO [89,90,91,92,93,94,95,96,97]. These results show that the doped ZnO is a good candidate for a CO sensor. Even though selectivity plays a vital role in a sensor’s performance, to date, DFT selectivity analyses of doped ZnO for CO detection are scarce. Therefore, future research should further investigate the selectivity of doped ZnO for CO gas detection.

## 4. Combined DFT and Experimental Investigations of Doped ZnO-Based CO Sensors

In the previous sections, experimental and theoretical contributions have been analyzed and discussed separately. However, because toxic gas sensing is a multidisciplinary field involving different topics, various new ZnO-based CO sensors should undoubtedly be found via combined theoretical–experimental studies to satisfy different demands. Hence, the search for new doped ZnO-based CO sensors remains a priority for experimentalists and theoretical researchers. In this sense, it has been shown that combining experimental results and DFT calculations is a good strategy for designing novel nanomaterials [98,99]. As a result, the design of new doped ZnO-based CO sensors has expanded from experimental findings to DFT calculations and vice versa.

**Figure 8:**
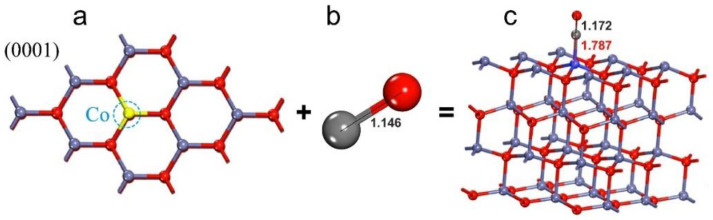
CO adsorption on the Co-doped ZnO (0001) surface. (**a**) Co-doped ZnO (0001) surface. (**b**) CO molecule, (**c**) CO adsorption on the Co-doped ZnO (0001) surface. Note that distances are in Å. Modified from Reference [100].

**Figure 9:**
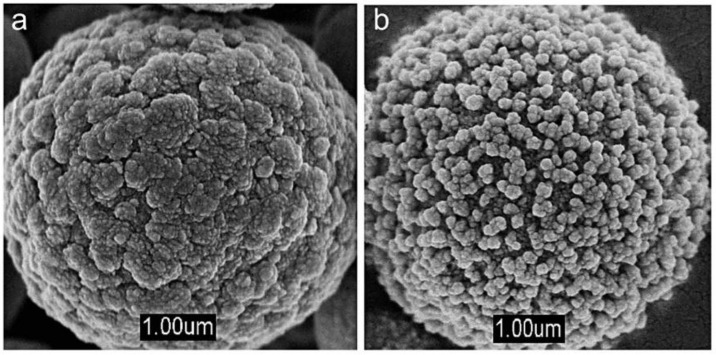
SEM images of bare (**a**) and Co-doped (**b**) ZnO. Obtained from Reference [100].

In this context, there are some combined theoretical and experimental studies on doped ZnO-based CO sensors [100,101]. Thereby, there are two ways to conduct combined theoretical and experimental studies on doped ZnO-based CO sensors. First, DFT calculations are used to interpret the experimental results at the molecular level. Second, DFT computations are used as a predictive tool for the rational design of novel gas sensors. These are subsequently validated by experimental techniques. In this direction, a combined theoretical–experimental study was conducted to investigate the Co-doped ZnO (0001) surface for CO sensing [100]. First, CO adsorption on the Co-doped ZnO (0001) surface was investigated using the PBE functional (Figure 8). The CO adsorption energy on the Co-doped ZnO (0001) surface was −2.31 eV. The adsorption energy and structural change (Figure 8c) of CO indicated that it was adsorbed via chemisorption interactions. Hence, such results suggest that the Co-doped ZnO (0001) surface is a good candidate for CO detection. Consequently, CO sensing experimental measurements were conducted to validate the theoretical results. Co-doped ZnO structures were synthesized using the hydrothermal method. As shown in Figure 9, Co doping increased the specific surface area and gas transport channels. These properties favor electron exchange between the gas and the sensor surface. Likewise, experimental findings demonstrated that the Co-doped ZnO is a good sensor for detecting CO gas [100].

**Figure 10:**
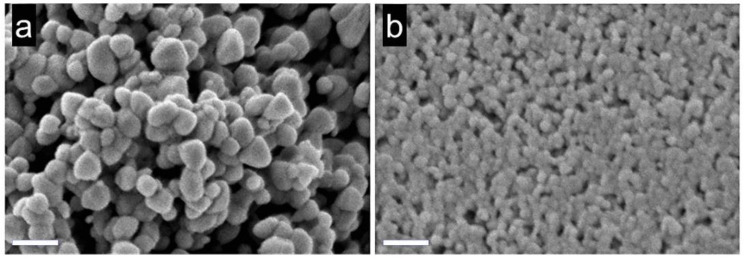
FE-SEM images for (**a**) ZnO (pure) (**b**) 0.5 wt. % Cr/ZnO. Note that scale bars: 100 nm. Obtained from Reference [101].

Another combined experimental–theoretical study investigated the use of Cr-doped ZnO nanostructures for CO sensing [101]. First, the Zn_12_O_12_ and Cr-doped Zn_11_O_12_ cluster properties were investigated using the B3LYP functional. Cr doping changed the structural, optical, and electrical properties of the Zn_12_O_12_ cluster. The energy bandgap of the Zn_12_O_12_ cluster (4.14 eV) significantly decreased compared with that of the Cr-doped Zn_11_O_12_ cluster (1.68 eV), suggesting that the Cr-doped Zn_11_O_12_ cluster presents a higher reactivity to the CO gas than that of the Zn_12_O_12_ cluster. Afterward, these theoretical results were experimentally corroborated. For this purpose, the sol-gel method was used to produce the undoped ZnO and 0.5 wt. % Cr-doped ZnO nanopowders. Subsequently, these nanopowders were used to obtain ZnO and 0.5 wt. % Cr-doped ZnO films. The average crystallite size of the undoped ZnO films (49.80 nm) significantly decreased compared with that of the 0.5 wt. % Cr-doped ZnO films (20.04 nm). The undoped ZnO and the 0.5 wt. % Cr-doped ZnO films have spherical particles at the nanometer scale; see Figure 10. The particle diameter distributions were ~40 nm for the undoped ZnO and ~25 nm for the 0.5 wt. % Cr-doped ZnO. This diminution in the particle size of the Cr-doped ZnO enhances the specific surface area. Thus, the sensors were evaluated in the presence of N_2_ as the carrier gas and CO as the test gas in a flow rate range of 300–500 sccm at 50 °C. The 0.5 wt. % Cr-doped ZnO sensor had a higher sensitivity to CO than that of the undoped ZnO sensor. For example, with a flow rate of 500 sccm, the 0.5 wt. % Cr-doped ZnO presented a sensibility of 65.45%, whereas the undoped ZnO presented a sensibility of 9.65%. In addition, Cr doping causes a substantial decrease in the response and recovery time. Therefore, the 0.5 wt. % Cr-doped ZnO is a better sensor than the undoped ZnO [101].

## 5. Conclusions

This review presents a detailed and critical analysis of the recent progress on doped ZnO for CO gas sensing. Based on this review, we conclude the following:

One important sensing parameter is the operating temperature, which is generally reported as ranging to greater than 300 °C for the undoped ZnO. However, transition metal-doped ZnO particles significantly reduce the operating temperature; the reported range is about 95 °C–180 °C for CO concentrations between 15 and 100 ppm. Low doping concentrations of transition metals between 1 and 5 at. % were used. These are considered the solubility limit because particle agglomerates are created at higher concentrations, which reduce the surface area.

Gas sensors doped with boron group metals exhibit improved response and recovery times of approximately 7–20 s and 16–45 s, respectively. The highest sensitivity was optimized under a CO atmosphere between 50 and 1750 ppm and an operating temperature ranging from 100 °C to 350 °C. In addition, the solubility reported was approximately 3 at. %, which is relativity low in contrast with the limit for the transition metals.

In the case of alkaline earth metals, the operating temperatures reported were 350 °C and 400 °C. These temperatures are higher than those of the transition metals, suggesting a disadvantage in the doped ZnO. The doping concentration was 5 at. %, similar to that of the transition metals. These findings open the door to exploring other alkaline metals (group II elements) to improve the characteristics of doped ZnO and enhance the sensitivity, selectivity, and reproducibility of ZnO gas sensors.

Selectivity plays an essential role in the performance of a sensor. However, to date, at the DFT level, analyses of the CO gas selectivity of doped ZnO are scarce. Therefore, future research should focus on the selectivity of doped ZnO for CO gas detection.

Combining theoretical and experimental studies is a good strategy for designing more sensitive and selective doped ZnO-based CO gas sensors. However, in the last five years, these types of research works have been scarce. Therefore, studies combining theory and experiment should be done to design novel CO gas sensors based on doped ZnO.

We still notice the need to continue exploring ZnO doping in search of novel CO sensors to improve the detection performance, repeatability, and stability, considering factors such as the crystal structure, oxygen vacancy content, and bandgap. This is important for CO monitoring as this gas is toxic even at low concentrations.

## Figures and Tables

**Table 1:** The main parameters of a gas sensor.

Parameters	Description
Response	It is defined as a change in some physical properties when the device is exposed to target species.
Selectivity	It is the ability of a gas sensor to detect high sensitivity to a specific gas among various types of gases at the same concentration level.
Sensitivity	It is referred in the graph where slope represents the correlation between gas response and the partial pressure of target gas.
Limit of detection	It is the lowest and highest concentration of the target gas that the sensor can detect.
Limit of detection	It is the highest gas concentration that the sensor can detect.
Operating temperature	It refers to the maximum temperature at which the device exhibits its maximum sensitivity in the presence of a target gas.
Repeatability	It is the response cycles of a sensor to be exposed to an analyte gas flow for a long time.
Response time	It is usually defined as the time it takes for gas sensor to respond to a concentration change.
Stability	It is the ability of gas sensors to conserve the output response measurement by a period, the level concentration of gas (ppm) unchanged.
Recovery time	Time measured when the gas sensor response changes in the interval of 90% to 10% when the sensor is exposed to a full-scale concentration of the gas, implying that the sensor exhibits 90% of the saturation value of resistance in seconds.

**Table 2:** Adsorption energies of CO on doped ZnO.

Material	E_ads_ (in eV)	Functional	Approach	Ref.
Al	−1.24 ^a^, −0.71 ^b^	B3LYP	Cluster (24 atoms)	[89]
Al	−0.79	PBE	Triangular nanowire (132 atoms)	[90]
Al	−1.12	PBE	Slab	[91]
In	−0.96 ^a^, −0.48 ^b^	B3LYP	Cluster (24 atoms)	[92]
In	−1.30	PBE	Slab	[93]
Pt	− 3.54	PBE	Cluster (24 atoms)	[94]
Sc	−0.86	B3LYP	Cluster (24 atoms)	[95]
Ti	−1.44	B3LYP	Cluster (24 atoms)	[95]
V	−1.67	B3LYP	Cluster (24 atoms)	[95]
Cr	−2.80	B3LYP	Cluster (24 atoms)	[95]
Mn	−1.45	B3LYP	Cluster (24 atoms)	[95]
Fe	−1.79	B3LYP	Cluster (24 atoms)	[95]
Cu	−1.01	PBE	Triangular nanowire (132 atoms)	[90]
Ga	−0.61 ^c^	B3LYP	Cluster (24 atoms)	[96]

The ^a^ is ΔH and ^b^ is ΔG, ^c^ the reported value was obtained from the text of the article.

## Data Availability

Not applicable.
